# Development of a food anaphylaxis education program to help parents live well with food allergies

**DOI:** 10.1186/1710-1492-7-S2-A4

**Published:** 2011-11-14

**Authors:** Nancy Ross, Cathy Gillespie, Nestor Cisneros, Allan Becker

**Affiliations:** 1 Thompson Children’s Allergy and Asthma Centre, Children’s Hospital, Winnipeg, Manitoba, Canada

## Background

Risk for food induced anaphylaxis appears to be increasing with significant impact on children, parents and family life. Families need to understand how to avoid the allergen and recognize and respond to reactions. Safety measures and risk must be balanced to ensure the child and family live well with the allergy. We developed and piloted an education program for parents of children <6 years old recently diagnosed with a severe food allergy.

## Method

• Literature review

• Creation of program objectives and outline

• Consultation with health care experts, parent representatives of food allergy organizations and an education specialist

• Development of program content and process

• Feedback from reviewers

• Piloted Program: participant pre-assessment, small group sessions, participant feedback post program

## Results

• We developed a small group, 2 session, interactive program using SMARTBoard^™^ technology, iclickers^®^, discussion, video clips, skill training and problem-solving

• 43 families registered

• 29 (67%) families completed ( 27 mothers, 16 fathers, 1 grandmother)

• Parental burden & self-efficacy pre-assessment indicated concerns about activities, communication and allergic reactions

• Auto-injector pre-assessment – 48 demonstrations observed; 73% did not complete all steps correctly

• 32 (73%) people completing the program believed 2 sessions were sufficient

## Conclusions

Our pilot suggests this interactive, small group program is a useful resource for families learning how to live with a serious food allergy. We identified activities and topics requiring adjustment and obtained feedback to help improve future sessions. Follow up with families will be important for further evaluation.

